# Direct observation of hydrogel network formation during sonication of cellulose dispersions

**DOI:** 10.1016/j.ultsonch.2026.107974

**Published:** 2026-07-22

**Authors:** Žan Boček, Tilen Kopač, Aleš Ručigaj, Matevž Dular

**Affiliations:** a Faculty of Mechanical Engineering, University of Ljubljana, Askerceva 6, 1000 Ljubljana, SI, Slovenia; b Faculty of Chemistry and Chemical Technology, University of Ljubljana, Vecna pot 113, 1000 Ljubljana, SI, Slovenia

**Keywords:** Cavitation, Cellulose nanofibrils, Sonication, Hydrogel network, Visualization

## Abstract

Nanocellulose dispersions and hydrogels represent a promising class of sustainable soft materials with dynamic responsiveness to external stimuli. This study employs visualization, statistical analysis, and particle image velocimetry to investigate the dynamic behavior of cationic (CCNF) and TEMPO-oxidized (TOCNF) cellulose nanofibril dispersions under sonication, focusing on hydrogel network formation at varying concentrations (0.5–2.0 wt% for CCNF and 1.0–2.0 wt% for TOCNF). CCNF dispersions rapidly formed physically crosslinked layers even at low concentrations due to attractive interactions via quaternary ammonium groups, resulting in persistent hydrogel networks. In contrast, TOCNF required concentrations over 1.3 wt% to overcome electrostatic repulsion between carboxylate groups and formed weaker, transient gels. Image sequence analysis revealed that crosslinked layer thickness and lifetime increased with concentration for both nanocellulose types, with 2.0 wt% samples exhibiting robust, resilient hydrogel structures throughout sonication. While CCNF showed resistance to ultrasonic disruption, TOCNF networks degraded rapidly due to weaker intermolecular interactions. Results highlight how nanofibril surface chemistry and concentration govern the interplay between ultrasound-induced network formation and its destabilization. The study provides mechanistic insights into sonication-driven gelation and establishes a methodological framework for designing nanocellulose-based materials with tailored real-time structural responsiveness, bridging nanostructural dynamics and macroscopic behavior for advanced soft material applications.

## Graphical abstract

**Figure:**
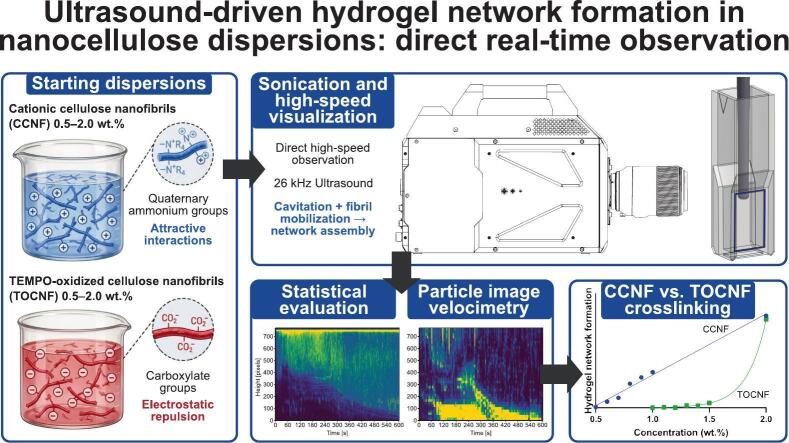

## 1 Introduction

Cellulose, the most abundant biopolymer on Earth, consists of linear chains of β-(1 → 4)-linked D-glucose units that aggregate into microfibrils through extensive hydrogen bonding [1]. These microfibrils form the structural basis of natural fibers and can be extracted, processed, or chemically modified into various nanostructured forms [2], most notably, cellulose nanocrystals (CNCs) [3] and cellulose nanofibrils (CNFs) [4]. These nanocellulose types differ not only in morphology [5] but also in physicochemical behavior [6] and potential applications [7]. In aqueous systems, cellulose can be dispersed to form colloidal suspensions or dispersions, depending on its processing history and structural form [8]. CNCs are typically rod-shaped, rigid particles obtained through acid hydrolysis, which removes the amorphous regions of cellulose [9]. CNFs, in contrast, are long, entangled fibers produced by mechanical or chemical fibrillation processes and exhibit high flexibility and entanglement capability [10]. These structural differences strongly influence how nanocellulose interacts with external stimuli such as shear or ultrasound [8].

Nanocellulose-based hydrogels are attracting growing attention in food applications due to their biocompatibility, biodegradability and tunable rheological properties [11], [12], [13], [14], [15]. This makes them promising materials for edible films and coatings, texture modification applications and food encapsulation. In latter case, nanocellulose hydrogels can improve stability and control the release of active compounds [11], [12]. In edible films they can offer improved mechanical strength, physical protection and moisture retention, leading to enhanced food preservation [13], [14]. Additionally, nanocellulose hydrogels are increasingly used as rheological modifiers, tailoring viscosity, texture and stability in food systems [15]. However, several challenges remain, including achieving precise gelation control, maintaining structural stability and balancing mechanical strength with desired swelling and release characteristics.

The application of ultrasound to aqueous nanocellulose dispersions has demonstrated markedly different effects depending on the nanocellulose type. Although CNCs are generally more structurally robust compared to CNFs due to their high crystallinity and rigidity [16], their behavior under sonication remains strongly dependent on processing conditions [17]. Recent studies have shown that carefully controlled sonication can improve dispersion quality by disrupting aggregates, modifying surface properties such as ζ-potential [18] and even inducing slight reductions in particle length under prolonged or high-intensity conditions [19]. These findings suggest that although CNCs are structurally more robust, they are not entirely immune to ultrasonic effects, particularly at the dispersion level [20], whereas their mechanical and flow properties remain largely unaffected by sonication [21], in contrast to the pronounced changes observed in CNF systems [22], [23].

In CNF-based dispersions, the mechanical energy input promotes fiber fragmentation, enhances dispersibility, and may stimulate gelation through increased surface interaction. This behavior positions CNFs as excellent candidates for the ultrasound-assisted formation of hydrogel networks [24]. Among CNFs, cationic cellulose nanofibrils (CCNF) and TEMPO-oxidized cellulose nanofibrils (TOCNF) are particularly promising due to their distinct surface charges. CCNF possess quaternary ammonium groups that introduce a permanent positive charge, enabling attractive interactions that facilitate rapid gelation [25]. In contrast, TOCNF carry carboxylate groups that render them negatively charged, stabilizing dispersions through electrostatic repulsion and requiring higher concentrations or external triggers to initiate gelation [26].

Ultrasonic treatment has emerged as a powerful tool for modifying cellulose-based systems. The cavitation phenomena induced by sonication produce intense local shear forces and microjets, which can enhance dispersion, reduce fibril aggregation, and increase the availability of surface functional groups by fragmenting nanofibrils into smaller units [27]. These processes facilitate hydrogen bonding and other secondary interactions between fibrils, ultimately supporting hydrogel network formation [28]. Indeed, recent studies [23], [29], [30], [31] have shown that prolonged or high-amplitude sonication increases the crosslink density of nanocellulose-based hydrogels, resulting in improved mechanical and flow properties such as viscosity and shear modulus.

The properties of nanocellulose-based hydrogels are strongly affected by the characteristics of the cellulose nanofibrils. These include fibril morphology, concentration and surface functionality. Modifications to the latter change the balance between attractive and repulsive intermolecular forces, thereby influencing network formation, crosslink density, swelling behavior, mechanical strength and rheological performance [8], [23], [25], [26]. Consequently, understanding how different nanocellulose surface chemistries affect hydrogel formation under unique processing conditions is essential for tailoring material performance to specific applications.

The effect of ultrasonic treatment on nanocellulose strongly depends on processing parameters such as frequency, amplitude, power density and sonication duration. These namely determine the cavitation intensity and resulting mechanical forces on the nanofibrils. Low-frequency, high-intensity ultrasound typically generates stronger cavitation, which promotes fibril fragmentation, dispersion, deagglomeration, enhances surface exposure and intermolecular interactions [23], [29], [30], [31]. Meanwhile prolonged sonication or excessive power may lead to over-fragmentation and deterioration of the nanocellulose network [27], [30]. Previous studies have shown that ultrasonic treatment can reduce aggregate size, modify surface charge and alter rheological properties in both cellulose nanocrystal and nanofibril systems [18], [19], [22], [30]. Consequently, careful consideration of ultrasonic parameters is essential for achieving an optimal balance between dispersion enhancement and preservation of network stability.

Most prior work has focused on structural characterization after sonication, after structural equilibrium has been reached [32], [33], or on bulk rheological measurement during or after processing [34]. Although these approaches provide important insight into dispersion behavior and gelation kinetics, they offer limited information on the spatially resolved dynamics of network formation and disruption during sonication. As such, the real-time dynamic processes that occur during sonication, such as network formation, reorganization or degradation mechanisms, remain largely unexplored. Particularly the knowledge regarding how differences in nanofibril surface chemistry govern real-time evolution of hydrogel structures under ultrasonic treatment is limited. This gap in knowledge is particularly critical when designing in situ responsive systems, where understanding the time-resolved evolution of gelation is essential [35]. Furthermore, the direct impact of nanofibril surface chemistry on sonication-induced network formation, degradation and reorganization has yet to be systematically evaluated [18].

To address these limitations, the present study provides a direct, real-time visualization of hydrogel network formation in CCNF and TOCNF dispersions during sonication. We hypothesize that the interplay between nanocellulose surface functionality, concentration, and sonication intensity critically governs the spatiotemporal dynamics of network formation, leading to distinct assembly pathways for cationic and anionic systems. Using visualization in combination with statistical analysis and particle image velocimetry (PIV), we examine this hypothesis by analyzing how these parameters influence hydrogel structuring under ultrasonic stimulation. The results reveal key differences in network behavior between cationic and anionic nanocellulose and provide new mechanistic insight into the reversible and irreversible processes that occur during acoustic exposure. By bridging the gap between molecular-level dynamics and macroscopic structure evolution, this work lays the foundation for better control and design of nanocellulose hydrogels in dynamic processing environments.

Unlike previous studies that characterize nanocellulose hydrogels only after sonication at structural equilibrium, this work uniquely captures the transient, real-time dynamics of network formation and disruption during active ultrasonic processing. Furthermore, by systematically comparing two chemically distinct nanocellulose types (CCNF and TOCNF) under identical sonication conditions, it directly isolates the role of surface chemistry in governing gelation pathways, which is an aspect not addressed in prior work.

## 2 Experimental set-up

### 2.1 Materials

Two nanocellulose modification types, cationic cellulose nanofibrils (CCNF) and TEMPO-oxidized cellulose nanofibrils (TOCNF), were studied. CCNF were sourced from Cellulose Lab, Canada (catalogue code: CNF-Cationic). According to the manufacturer, CCNF was produced from pulp fibers via nano-fibrillation using a high-pressure homogenizer. The nanofibrils carry quaternary ammonium groups on their surface (containing Cl⁻ as counter ions), resulting in a cationic charge density of 2.4 mmol/g, while the presence of hydroxy groups contributes to their surface hydrophilicity. TOCNF with the chemical composition [(C_6_H_10_O_5_)_x_(C_6_H_9_O_4_COONa)_γ_] and a carboxylate content of 1.5 mmol/g (based on dry solids) (containing Na^+^ as counter ions), were obtained from Cellulose Lab (Canada) (catalogue code: CNF-Anionic).

Structurally, the CCNF and TOCNF exhibit a network-like morphology with an average width of approximately 50 nm and lengths extending up to several hundred micrometers. Their hydrophilic surface area, as measured by BET analysis, ranges from 31 to 33 m^2^/g. The material was supplied as a 3 wt% aqueous gel with a density of 1.0 g/cm^3^, while the dry powder form has a density of 1.5 g/cm^3^. Both nanocellulose types are derived from fully bleached pulp and are therefore composed of nearly pure cellulose (>95 wt%), with only trace amounts of hemicellulose and negligible lignin.

### 2.2 Nanocellulose sample preparation

Experiments were conducted using 0.5, 0.6, 0.7, 0.8, 0.9, 1.0, 2.0 wt% and 1.0, 1.1, 1.2, 1.3, 1.4, 1.5, 2.0 wt% aqueous CCNF and TOCNF solutions, respectively. These concentrations were chosen based on preliminary experiments (data not show), which found that observable gelation occurs above 0.5 wt% for CCNF and above 1.0 wt% for TOCNF solutions. A 3.0 wt% aqueous TOCNF dispersion was prepared by gradually adding demineralized water containing 0.02 wt% sodium azide (to prevent microbial contamination) to freeze-dried TOCNF powder. The resulting non-homogeneous dispersion was stirred at 750  rpm using a four-blade propeller stirrer for 1  h. The sample had a pH value of 7. To improve dispersion, the solution was then treated in an Iskra PIO Sonis 4GT ultrasonic bath at a constant 20 ± 1°C for 1  h at a frequency of 30 kHz and 400 W of power. Subsequently, the sample was stirred overnight at 750  rpm at room temperature to ensure complete homogenization.

Lower concentrations of CCNF and TOCNF dispersions were made by diluting the mother 3 wt% solutions with appropriate amounts of demineralized water. For the PIV analysis, glass spheres (≈1.1 g mL^−1^) with diameters between 9 and 13 µm were additionally dispersed into the solutions at an approximately concentration of 0.4 mg mL^−1^. The solutions were homogenized using a vortex mixer (F202A0175 IR Vortex Mixer, Velp Scientifica, Italy) at approximately 2000 rpm. They were again mixed immediately before visualization experiments.

### 2.3 Nanocellulose sonication

**Fig. 1:**
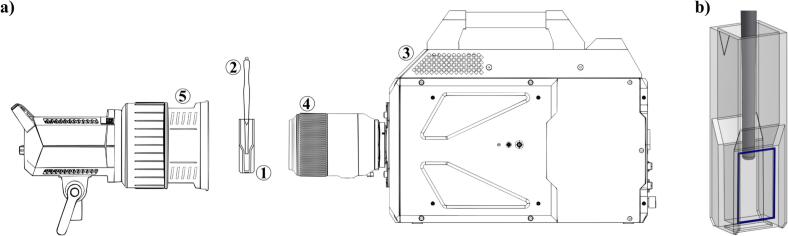
Experimental set-up for observation of hydrogel network formation: (a) equipment layout and (b) detailed view of sonotrode position inside the cuvette. The blue outline in the latter represents the camera’s field-of-view.

The experimental set-up for sonication and visualization is shown in Fig. 1. All experiments were conducted inside standard semi-micro spectroscopy cuvettes (1) at 25°C. The sample’s temperature was controlled using a temperature probe and two aluminum heat exchangers. They were mounted to the cuvette’s sides and connected to a high-power chiller. The cuvettes were filled with 1.5 mL of respective sample. A Hielscher UP200St ultrasonic homogenizer was used for sonication, equipped with a S26d2 sonotrode – 2 mm tip diameter (2). The ultrasonic horn (UH) was immersed into the sample approx. 7 mm below liquid level, so to be visible in the lower sections of the cuvette (Fig. 1b, blue outline). The homogenizer operated at a frequency of 26 kHz and a constant 20% amplitude and power output of approx. 8 W for all experiments.

### 2.4 Visualization

A Photron FASTCAM SA-Z high-speed camera (3) was used for visualization of the dynamic response of cellulose dispersions under sonication (Fig. 1a). Although the camera is capable of up to 60 000 frames per second at this resolution, it was used due to its high shutter speed and light sensitivity. Its recording speed was set to 125 frames per second and its shutter speed to 2.48 × 10^-7 s^. The recording resolution was adjusted to 768 × 472 pixels, such that only the lower sections of the cuvettes were visible in the camera’s field of view (Fig. 1b, blue outline). The camera was equipped with a 105 mm Nikon NIKKOR AF-S VR lens (4), with the focal length set to its minimum of 31.4 cm. This resulted in a pixel size of 47 pixels mm^−1^ or the entire field of view being 16.3 × 10.0 mm. Lighting was provided with a 200 W Godox Litemons LA200D LED light (5), positioned behind the sample cuvette opposite the high-speed camera (Fig. 1a). The LED light’s power output during each experiment was adjusted based on nanocellulose solution transparency. The sonication and visualization sequence for each sample lasted 10 min, with the ultrasonic homogenizer operating continuously. Sonication experiments and the consequent statistical analysis of each nanocellulose type and concentration have been repeated at least three times. The below presented results show a representative example from each sample group.

### 2.5 Image sequences analysis

**Fig. 2:**
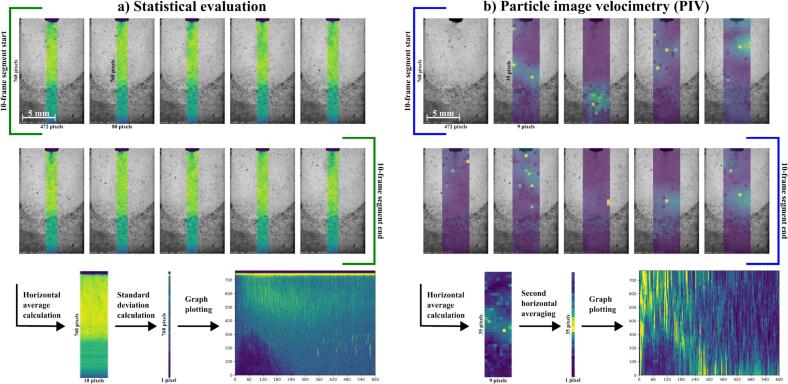
Schematic representation of image sequences analysis: (a) statistical evaluation and (b) particle image velocimetry (PIV).

The visualization results were stored and analyzed in segments of 10 frames. Both the statistical evaluation (Fig. 2a) and PIV (Fig. 2b) were conducted using custom-made Python scripts (version 3.12.7) with the pyMRAW [36] and openPIV [37] libraries.

#### 2.5.1 Statistical evaluation

A schematic representation of the statistical evaluation of sonication sequences is shown in Fig. 2a. It was performed by first calculating the horizontal pixel average of an 80-pixel wide band from the middle of each frame. These were then combined within each 10-frame segment and used to calculate the standard deviation within each segment. The resulting standard deviation values were used to plot the graphs for sample behavior during sonication. In these graphs the darker areas represent less or no solution movement, while the brighter areas (as for example near the tip of the sonotrode) indicate faster movement.

The results of statistical sonication evaluation were further analyzed to determine the boundaries between physically crosslinked and non-crosslinked areas of each sample. This was then used to calculate the crosslinked layer height throughout sonication. The resulting data enabled a more concise comparison of sonication behavior based on nanocellulose modification type and solution concentration.

#### 2.5.2 Particle image velocimetry (PIV)

Due to the slower speeds of solution movement during sonication, the PIV analysis was conducted for every 10th segment of the visualization results. The analysis parameters were set to a 32-pixel interrogation windows size, a 38-pixel search area size and a 47-pixel mm^−1^ scale. Velocity fields for image pairs were thus calculated using 200-pixel wide bands from the middle of the frames – 9 pairs per segment (Fig. 2b). The horizontal averages of these velocity fields were then calculated and combined within the 10-frame segment. For the second time, horizontal averages of these new values were calculated and then combined into graphs. As with the statistical evaluation results, the darker areas here represent lower calculated velocities, i.e. slower sample movement during sonication, and vice versa. The color bar only indicates crosslinking on a relative scale from zero to one, with zero being the most crosslinked (slowest movement). The highest measured velocities (yellow areas on Fig. 5b) ranged up to approx. 1.2 m s^−1^.

## 3 Results and discussion

The following section presents the results on the influence of sonication on the dynamic behavior of aqueous CCNF and TOCNF dispersions, which can progressively evolve into hydrogel networks over time. Previous studies have repeatedly shown that sonication of cellulose fibrils induces gel-like behavior. In our recent work [23], we examined the rheological properties of CCNF- and TOCNF-based hydrogels as a function of sonication amplitude and duration. In the present study, however, we focus for the first time on elucidating the in situ dynamic behavior of cellulose fibrils during sonication. To this end, all experiments were conducted under constant and well-controlled constant sonication conditions.

### 3.1 Direct observation of dynamic cellulose dispersion response to sonication

**Fig. 3:**
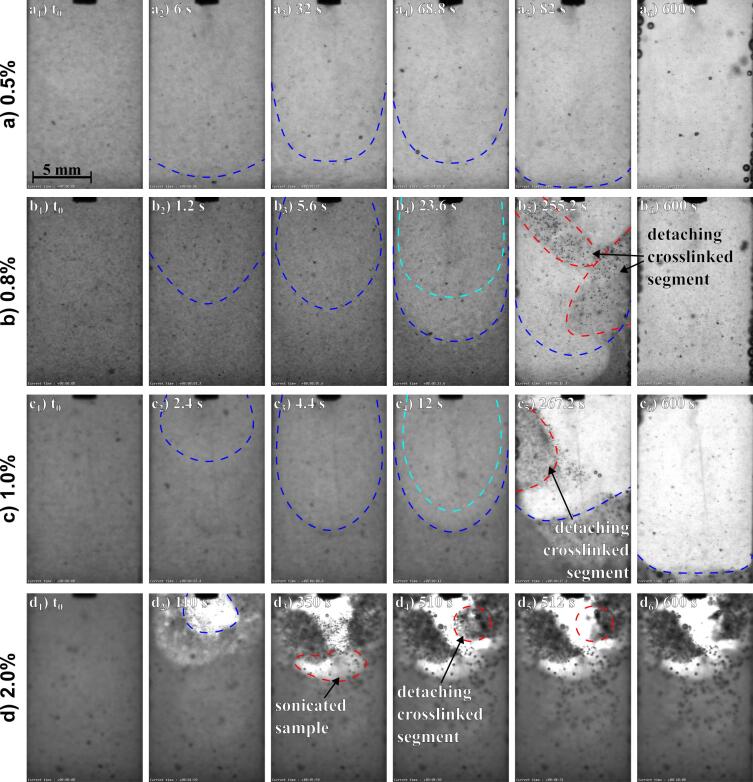
Sequences showing the dynamic responses of (a) 0.5, (b) 0.8, (c) 1.0 and (d) 2.0 wt% cationic cellulose nanofibril (CCNF) samples to sonication (Movies in supplementary). The blue dotted lines show the edge of the strongly physically crosslinked layer, cyan lines show the edge of the buffer layer and red lines show other significant observations. For better insight into the marked phenomena, readers are encouraged to view the supplementary video materials.

**Fig. 4:**
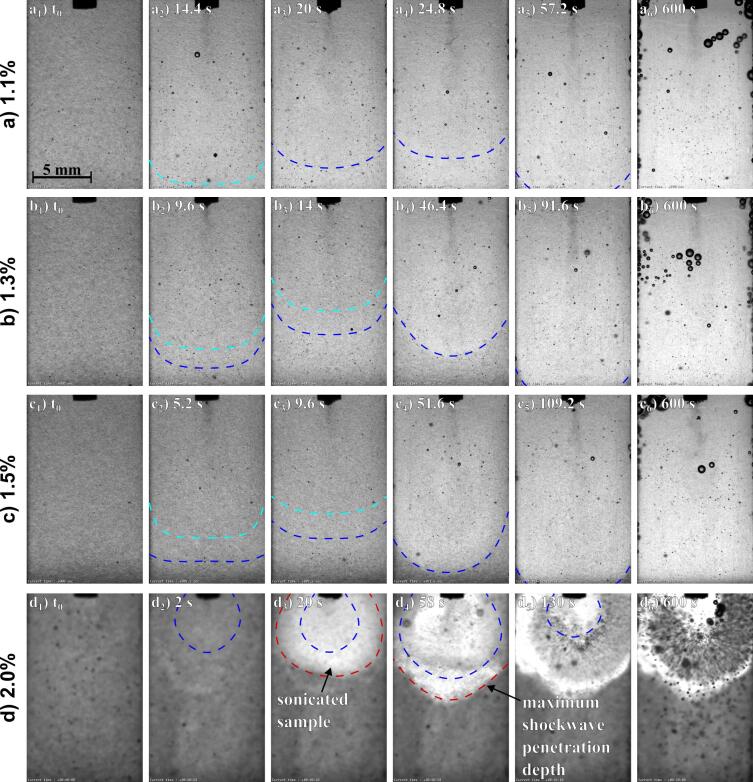
Sequences showing the dynamic responses of (a) 1.1, (b) 1.3, (c) 1.5 and (d) 2.0 wt% TEMPO-oxidized cellulose nanofibril (TOCNF) samples to sonication (Movies in supplementary). The blue dotted lines show the edge of the strongly crosslinked layer, cyan lines show the edge of the buffer layer and red lines show other significant observations. For better insight into the marked phenomena, readers are encouraged to view the supplementary video materials.

This section details the sonication behavior of CCNF and TOCNF at varying concentrations, highlighting differences in hydrogel network formation and general sonication trends. CCNF’s cationic quaternary ammonium groups promote rapid gelation through attractive interactions, while TOCNF’s anionic carboxylate groups induce electrostatic repulsion, requiring higher concentrations for network formation. To fully appreciate the dynamic nature of these processes, readers are encouraged to view supplementary video materials, which provide visual insights into the sonication sequences. Selected sequences are presented in Fig. 3, Fig. 4 below, with the complete set of visualization results available in supplementary materials. In each sonication sequence, the first and last frames represent the initial and final states of the sample during sonication, respectively.

**Fig. 5:**
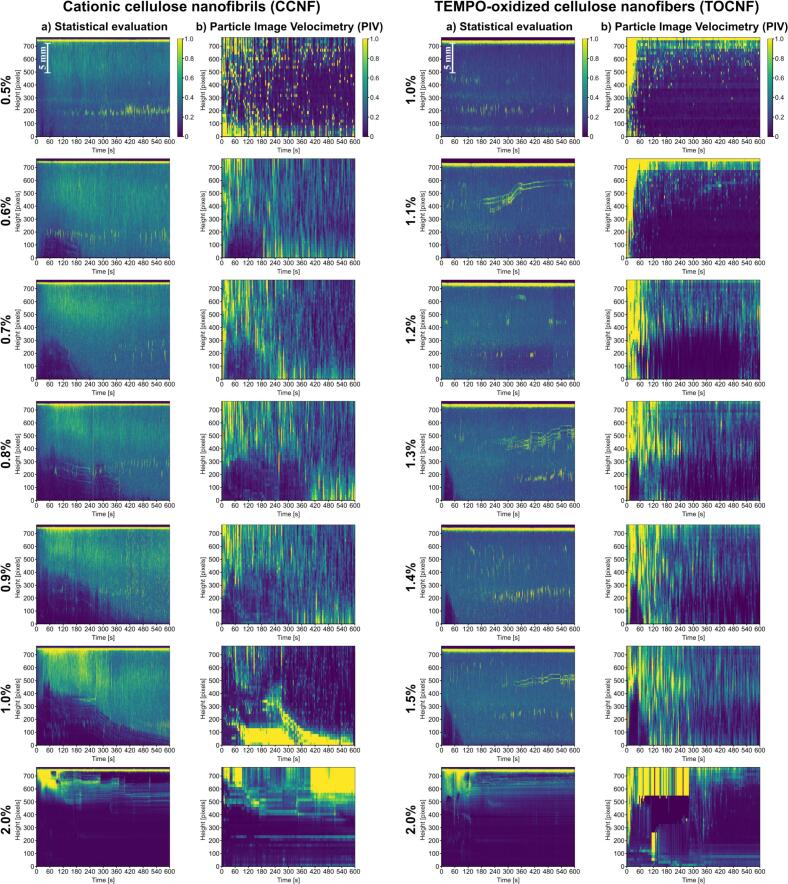
Image sequence analysis of sonication visualization results: (a) statistical evaluation and (b) particle image velocimetry (PIV). The left two columns represent 0.5, 0.6, 0.7, 0.8, 0.9, 1.0 and 2.0 wt% cationic cellulose nanofibril (CCNF) samples, and the right two columns 1.0, 1.1, 1.2, 1.3, 1.4, 1.5 and 2.0 wt% TEMPO-oxidized cellulose nanofibril (TOCNF) samples. The color bar represents relative physical crosslinking from zero to one, with zero being the most crosslinked (slowest solution movement). The highest velocities measured using PIV ranged up to approx. 1.2 m s^−1^ (yellow sections).

#### 3.1.1 Dynamic evolution of physically crosslinked structures in CCNF systems under sonication

Sonication of CCNF solutions at concentrations of 0.5, 0.6, 0.7, 0.8, 0.9 and 1.0 wt% exhibited broadly similar behavior, with minor variations observed at the lowest concentration (0.5 wt%) due to weaker intermolecular interactions. Increasing CCNF concentration led to gradual increase in sample opacity across recordings, reflecting higher nanofibril density.

The sonication sequences for 0.5, 0.8 and 1.0 wt% CCNF solutions are shown in Fig. 3a–c. As the process began, a distinct, slower moving layer formed rapidly at the cuvette’s bottom (Fig. 3a2, b2 and c2). This behavior is consistent with enhanced interactions between nanofibrils, likely involving hydrogen bonding and electrostatic interactions, which are well-established as the primary drivers of physical gelation in nanocellulose systems [38], [39], [40]. The layer formation occurred earlier and grew thicker with higher concentrations, reflecting increased nanofibril density that facilitates network formation. Its reduced mobility compared to regions near the UH results from increased viscosity due to physical crosslinking. Periodic pressure waves penetrated this crosslinked layer (Fig. 3a3, b3 and c3), displacing segments outward toward the cuvette walls and upward toward the UH, where they disintegrated back into the non-crosslinked liquid phase. Consequently, the upper regions of the cuvette remained liquid, as stronger pressure waves and currents likely inhibited physical crosslinking by disrupting chain alignment.

Once a thicker, strongly crosslinked layer formed at the cuvette’s bottom (Fig. 3b4 and c4), ultrasonic pressure waves could no longer penetrate deeply into it, except in the 0.5 wt% CCNF solution. At this lower concentration, weaker physical crosslinking allowed pressure waves to continuously dislodge segments from the crosslinked layer, leading to its repeated reformation (Fig. 3a4). In contrast, for solutions at 0.6 wt% and higher, a distinct buffer layer emerged between the non-crosslinked solution near the UH, and the strongly physically crosslinked layer at the cuvette’s bottom (Fig. 3b4 and c4). This buffer layer exhibited slower movement compared to the non-crosslinked solution, flowing outward along the crosslinked layer and upward at the cuvette’s edges. After a certain amount of time, the buffer layer ceased moving, likely due to sufficient physical crosslinking that restricted its flow, and a new buffer layer formed it. This cycle of buffer layer physical crosslinking and replacement occurred more frequently in higher-concentration samples, reflecting increased nanofibril interactions.

Across all tested CCNF concentrations, an intermediate buffer layer formed between the non-crosslinked liquid and the physically crosslinked layer at the cuvette’s bottom. It gradually diminished and disappeared as crosslinking progressed, increasing viscosity and reducing mobility. The absence of the buffer layer allowed ultrasonic pressure waves to penetrate the strongly crosslinked layer at the cuvette’s bottom, causing larger segments to periodically detach (Fig. 3b5 and c5). These segments disintegrated near the UH into the non-crosslinked liquid phase. This shedding intensified over time, leading to progressive degradation of the physically crosslinked layer, which occurred more rapidly in samples with lower CCNF concentrations (Fig. 3a5, b5 and c5). No new crosslinking was noted throughout the solution, likely due to strong currents inside the non-crosslinked solution that prevented chain alignment and inhibited network reformation, even near the cuvette’s bottom. However, in the case of the 0.9 and 1.0 wt% CCNF solutions, part of the physically crosslinked layer persisted throughout the sonication process (Fig. 3c6). Higher concentrations likely enhanced physical crosslinking strength, enabling the polymer network to resist complete disintegration into the liquid phase.

The 2.0 wt% CCNF solution exhibited distinct sonication behavior compared to lower concentrations and warrants separate discussion (Fig. 3d). Here the difference between sonicated and non-sonicated solution was immediately apparent. The high CCNF concentration increased solution viscosity, limiting pressure wave penetration to the region near the UH (Fig. 3d2). A slow-moving buffer layer formed early in the sonication process between the non-crosslinked liquid and the strongly physically crosslinked layer at the cuvette’s bottom. This buffer layer quickly transitioned to weak oscillations, indicating restricted mobility (see Movie 7 in supplementary).

**Fig. 6:**
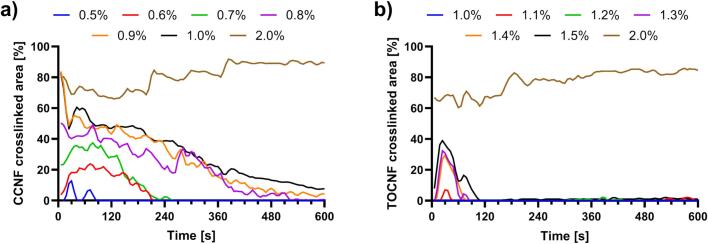
Relative area of physically crosslinked nanocellulose solution as a function of sonication time: (a) 0.5, 0.6, 0.7, 0.8, 0.9, 1.0, 2.0 wt% CCNF and (b) 1.0, 1.1, 1.2, 1.3, 1.4, 1.5, 2.0 wt% TOCNF.

As sonication continued, individual pressure waves unevenly penetrated deeper into the sample (Fig. 3d3). In rare instances, this caused individual crosslinked segments to separate and quickly disintegrate (Fig. 3d4 and d5). Apart from this, no other noteworthy mechanisms were observed, with pressure waves affecting approximately 45% of sample depth by the end of sonication (Fig. 3d6), indicating a robust physically crosslinked network that resisted extensive disruption. A comparison of crosslinked layer thickness as a function of time for all studied CCNF concentrations is shown in Fig. 6a.

#### 3.1.2 Gelation and dynamic reorganization in TOCNF under sonication

Compared to CCNF, the sonication effects on TOCNF solutions were evident only at higher concentrations due to strong electrostatic repulsion from anionic carboxylate groups, which hinder nanofibril interactions at low concentrations. No crosslinking was observed in the 1.0 wt% TOCNF sample. Significant physical crosslinking occurred at concentrations of 1.3 wt% and above (Fig. 4), with minimal crosslinking visible in the 1.1 wt% and 1.2 wt% solutions (Fig. 4a). In the 1.1 wt% TOCNF solution, a weakly crosslinked layer formed near the bottom of the cuvette after approximately 14 s of sonication (Fig. 4a2), as ultrasonic cavitation temporarily overcame repulsion to enable weak hydrogen bonding and ionic interactions. This layer thickened gradually (Fig. 4a3) but ceased growing shortly thereafter (Fig. 4a4) and soon afterwards disintegrated (Fig. 4a5), when cavitation-induced shear forces disrupted weak bonds. For the remainder of sonication, the solution behaved similarly to the 1.0 wt% TOCNF sample, with no visible crosslinking or other changes.

The sonication sequences for 1.3 and 1.5 wt% TOCNF solutions (Fig. 4b and c) showed physical crosslinking within seconds, forming a viscous layer near the cuvette’s bottom (Fig. 4b2 and c2) as ultrasonic cavitation enhanced hydrogen bonding and ionic interactions via carboxylate groups. The 1.4 wt% solution exhibited intermediate behavior. Higher nanofibril density reduced electrostatic repulsion, promoting earlier layer formation in more concentrated solutions, though time differences were minimal. A buffer layer, more viscous than the non-crosslinked liquid but less than the physically crosslinked layer, formed between these regions (Fig. 4b2 and c2). As sonication progressed, the crosslinked layer thickened rapidly, with the buffer layer maintaining its thickness and mobility (Fig. 4b3 and c3). Unlike with different CCNF concentrations, where physical crosslinking varied with concentration, the crosslinked layers in 1.3, 1.4 and 1.5 wt% TOCNF solutions reached their maximum thickness at similar times. Subsequently, the crosslinked layer began to degrade, at which time the buffer layer disappeared (Fig. 4b4 and c4). In later stages, ultrasonic pressure waves penetrated the crosslinked layer, dispersing it completely (Fig. 4b5 and c5). Afterwards, the 1.3, 1.4 and 1.5 wt% TOCNF solutions behaved similarly to the 1.0, 1.1 and 1.2 wt% samples post-disintegration, showing no further crosslinking due to acoustic streaming.

The 2.0 wt% TOCNF solution exhibited distinct sonication behavior due to high viscosity from dense nanofibril networks and electrostatic repulsion from carboxylate groups, limiting cavitation efficiency, as shown in Fig. 4d. Before sonication, the sample appeared uniform (Fig. 4d1). Upon initiation, sonication was immediately restricted to a small area directly underneath the UH (Fig. 4d2). During further treatment, sonication remained comparatively limited. However, the sonicated solution began to accumulate around this region and gradually expanded outward (Fig. 4d3). A slow-moving buffer layer quickly formed between the strongly crosslinked and non-crosslinked sections, transitioning to weak oscillations as partial physical crosslinking restricted mobility.

After approximately 55 s, pressure waves penetrated the crosslinked layer, causing significant displacement and disintegration of detached segments, expanding the sonicated area (Fig. 4d4). Penetration depth remained limited thereafter. The sonicated area continuously contracted to a smaller region near the UH, reaching its minimum at around 130 s (Fig. 4d5). Throughout the remainder of sonication, the previously sonicated region gradually increased in opacity (Fig. 4d6), suggesting nanofibril aggregation or network reorganization due to disrupted intermolecular interactions under prolonged shear forces. Fig. 6b shows the combined comparison of crosslinked layer thickness during sonification for all TOCNF samples.

Although both 2.0 wt% CCNF and TOCNF formed stable hydrogel networks, the denser crosslinked structure observed in CCNF suggests mechanical stability and lower swelling capacity compared to TOCNF. Previous studies have shown that increased network density in nanocellulose hydrogels generally results in higher mechanical strength and reduced water uptake [23], [25]. Meanwhile more open nanofibril networks exhibit greater swelling and lower rigidity. These findings further support the structural differences observed between CCNF and TOCNF in the present study.

### 3.2 Statistical and PIV analysis of CCNF and TOCNF sonication sequences

The results of image sequence analysis are presented in Fig. 5. Here the results for CCNF and TOCNF samples are placed in the left and right two columns, respectively. For each nanocellulose modification type, the (a) columns show the results of statistical evaluations – changes in pixel brightness at different heights during sonication. Lighter areas indicate dynamic motion in non-crosslinked liquid, while darker areas denote reduced or absent movement in physically crosslinked regions, where nanofibril interactions form stable networks. The (b) columns display PIV analysis, with brighter colors representing faster solution movement and darker colors indicating slower or static regions, consistent with physical crosslinking-induced viscosity increases.

For the CCNF samples (Fig. 5a, left), statistical analysis reveals a gradual increase in physically crosslinked regions near the cuvette’s bottom with higher nanofibril concentrations, as greater fibril density enhances hydrogen bonding and ionic interactions via quaternary ammonium groups, promoting network formation. This crosslinking is most pronounced in the initial minutes of sonication. Prolonged sonication, however, reduces the crosslinked layer’s thickness due to cavitation-induced shear forces disrupting intermolecular bonds. At concentrations below 1.0 wt%, the physically crosslinked layer fully disintegrates, reflecting weaker network stability, whereas at 0.9 and 1.0 wt%, portions persist (as noted in Section 3.1.1) due to stronger physical crosslinking from increased nanofibril interactions. The 2.0 wt% CCNF solution exhibits distinct behavior, with high viscosity limiting cavitation to the UH vicinity, reducing the extent of physical crosslinking and movement compared to lower concentrations.

The PIV analysis of CCNF sonication (Fig. 5b, left) confirms these trends, showing no distinct crosslinking in the 0.5 wt% sample due to insufficient nanofibril density for stable network formation. At 0.6 and 0.7 wt%, short periods of rapid movement occur across the cuvette height before physical crosslinking emerges, driven by initial cavitation-induced mixing. Although an increase in crosslinked layer height can be observed in the beginning of 0.8, 0.9 and 1.0 wt% solution sonication, some parts appear to be physically crosslinked from the very start. Noticeable is also a steady increase in crosslinked layer height and longevity with increasing CCNF concentration. This indicates increased physical crosslinking strength. Additionally, instead of a sharp transition between the faster moving liquid sections and the crosslinked layers, transitional layers are visible. These have slower speeds but are not completely static, which could indicate the buffer layers previously described in section 3.1.1. These layers arise from a balance between cavitation-induced shear and intermolecular bonding. Increasing the CCNF concentration to 2.0 wt% shows a complete change in sonication behavior (Fig. 5b, left). Due to the high concentration, sonication is visibly limited to the area around the UH. After approximately 90 s of sonication a sudden decrease in movement speed of the non-crosslinked solution is noticeable. Accompanying this is a gradual increase in the depth of the sonicated area. However, this increase stops after about 180 s, after which the depth remains constant. After approximately 420 s of sonication the speed of the non-crosslinked section abruptly increases again and remains at this level till the end of sonication.

Statistical and PIV analysis of TOCNF sonication sequences, shown in Fig. 5, right, reveal distinct behaviors compared to CCNF due to the anionic carboxylate groups on TOCNF, which induce electrostatic repulsion and hinder physical crosslinking at lower concentrations. The (a) column displays statistical evaluations of pixel brightness changes at different cuvette heights. Here lighter areas indicate dynamic movement in non-crosslinked liquid driven by ultrasonic cavitation, and darker areas denote physically crosslinked regions with reduced mobility due to network formation. The (b) column presents PIV analysis, where brighter colors represent faster movement and darker colors indicate slower or static regions, reflecting viscosity increases from physical crosslinking.

Statistical analysis (Fig. 5, right) highlights smaller physically crosslinked areas in TOCNF compared to CCNF, as electrostatic repulsion limits nanofibril interactions. As already discussed in section 3.1.2, no crosslinking occurs in the 1.0 wt% TOCNF sample due to insufficient nanofibril density to overcome repulsion. At 1.1 wt%, limited physical crosslinking, observed in visualization (Section 3.1.2), is confirmed, as cavitation temporarily enables weak hydrogen bonding. However, the 1.2 wt% sample shows no detectable crosslinking in statistical evaluation, likely due to sensitivity limitations in detecting transient networks. At 1.3, 1.4 and 1.5 wt%, physical crosslinking becomes evident within seconds, forming viscous layers near the cuvette’s bottom as increased nanofibril density reduces repulsion. These layers reach maximum thickness around 20 s, with similar timing across concentrations, suggesting a threshold where the gelation stabilizes. After this their thickness starts to immediately decrease until the layer completely disappears due to cavitation-induced shear forces breaking weak intermolecular bonds, disappearing faster than in the CCNF samples. Even at the highest concentration, the crosslinked layer disappears completely, unlike the persistent layers in 0.9 and 1.0 wt% CCNF, reflecting weaker TOCNF network stability. No further crosslinking is visible in any of the samples after the initial physically crosslinked layer disappears, as sonication prevents network reformation.

PIV analysis (Fig. 5b, right) mirrors these trends showing no distinct slower moving (physically crosslinked) sections at 1.0–1.2 wt% due to dominant repulsion. At 1.3–1.5 wt%, physical crosslinking forms rapidly, driven by cavitation-enhanced interactions. Unlike CCNF, where partial crosslinking may pre-exist, TOCNF samples show no initial crosslinking, as anionic repulsion requires active cavitation to initiate gelation. Transitional buffer layers, less distinct than in CCNF, appear between non-crosslinked and physically crosslinked regions, exhibiting slower movement due to partial physical crosslinking balancing shear and bonding forces (as discussed in section 3.1.2). At 2.0 wt%, high viscosity from dense nanofibril networks limits cavitation to the UH area, confining fast movement to the upper cuvette initially (Section 3.1.2). Statistical evaluation (Fig. 5a, right) shows a momentary expansion of the sonicated area at 55 s, likely due to a high-energy cavitation event, followed by contraction. PIV analysis indicates reduced movement after 130 s (statistical) and 270 s (PIV), suggesting viscosity-driven damping or aggregation. No further phenomena were observed, indicating a stable, viscous state.

### 3.3 Dynamic crosslinking behavior of modified nanocellulose during sonication

The effect of sonication on the mechanical and flow properties of cellulose-based systems has been recently explored [23]. It was hypothesized that sonication fragments cellulose fibers into smaller units, increasing their specific surface area and exposing more surface hydroxy groups for hydrogen bonding. This enhances crosslink density, leading to higher shear moduli, viscosities, and critical stresses, reflecting enhanced mechanical strength and altered flow behavior. Unlike prior work, where samples rested for 24 h post-sonication to reach structural equilibrium before measurements, this study focuses on dynamic processes during sonication, aiming to uncover the structural changes that drive these enhanced properties.

Statistical analysis of CCNF and TOCNF sonication sequences (Fig. 5a) enables comparison of relative physically crosslinked layer heights, presented in Fig. 6. This additionally enables the quantitative comparison of gelation gelation kinetics, maximum thickness and structural longevity during sonication. We hypothesized that sonication disrupts cellulose nanofibrils, thereby increasing their specific surface area and exposing a greater number of surface functional groups (e.g., hydroxy and quaternary ammonium in CCNF, carboxylate in TOCNF). These groups can participate in hydrogen bonding and other secondary interactions, facilitating the formation of a hydrogel network capable of swelling. Additionally, the mechanical energy from sonication mobilizes cellulose chains, bringing them into closer proximity to form intermolecular interactions that would otherwise be unlikely at rest. The result for both is an increase in crosslink density, leading to a more defined and robust hydrogel structure (see short times up to 100 s in Fig. 6).

However, prolonged sonication may disrupt these weak intermolecular interactions and cause partial network collapse. Notably, once sonication ceases, the network begins to reassemble, often achieving higher crosslink density due to increased availability of reactive surface groups. Furthermore, concentration plays a crucial role in governing the behavior of cellulose fibers during sonication. At lower concentrations, near the minimum threshold for hydrogel formation, limited intermolecular interactions lead to network disruption during prolonged sonication, causing temporary collapse. In such cases, the network re-establishes itself over time, typically within 24 h post-sonication as chains reorganize. In contrast, at higher concentrations, denser nanofibril networks withstand cavitation-induced shear, consistently enhancing crosslink density and reinforcing the hydrogel structure.

The distinct sonication behaviors of CCNF and TOCNF arise from their surface chemistry. At the molecular level, the different responses originate from the nature of their surface functional groups and the balance between attractive and repulsive interfibrillar interactions [8], [27]. The quaternary ammonium groups present on CCNF carry a permanent positive charge and promote interfibrillar associations through hydrogen bonding and ionic interactions, facilitating the formation of a compact and mechanically resilient network [23], [25]. In contrast, TEMPO oxidation introduces negatively charged carboxylate groups onto the TOCNF surface, increasing the surface charge density and generating electrostatic double-layer repulsion between neighboring fibrils. As a result, TOCNF fibrils remain more individualized and require higher concentrations to achieve sufficient fibrillar crowding for network formation [26]. Consequently, CCNF forms denser and more persistent physically crosslinked structures, whereas TOCNF forms more open and transient networks that are more susceptible to cavitation-induced shear and prolonged ultrasonic treatment [27], [30]. This difference explains why CCNF hydrogels can form at lower concentrations and maintain partial structural integrity during sonication, while TOCNF networks require higher concentrations and undergo complete disintegration at lower concentrations. At higher concentrations, both materials form denser networks; however, CCNF consistently exhibits greater crosslink density and network stability due to stronger interfibrillar associations [40].

Starting with 0.5 wt% CCNF, a shallow physically crosslinked layer distinctly appeared twice, at approximately 30 and 70 s, with the second layer reaching a lower maximum height, reflecting the minimal nanofibril density near the threshold of hydrogel formation. In the case of all the other, more concentrated solutions, a crosslinked layer was detected at the immediate beginning of sonication. Therefore, we conclude that the minimum concentration of CCNF required to form a swellable hydrogel network is approximately 0.5 wt%. At lower concentrations, the number of available hydroxy and other surface substituent groups is insufficient to establish a stable, swellable hydrogel network. The presence of two minor peaks, observed exclusively in this sample, further supports the hypothesis that at 0.5 wt% concentration, the hydrogel network is only beginning to form.

At 0.6 and 0.7 wt% CCNF, physically crosslinked layers form immediately upon sonication due to increased nanofibril density, reaching maximum heights of approximately 20 and 35 wt% of the visible solution, respectively (Fig. 6). After approximately 100 s of sonication, shear forces from collapsing cavitation bubbles cause the layers to thin, reaching comparable thicknesses by 150 s and disappearing completely at approximately 210 and 255 s, respectively, as weaker interactions are disrupted. At higher concentrations, the hydrogel network forms earlier and achieves a higher physically crosslinked area percentage, owing to the increased availability of surface substituent groups. Consequently, the network exhibits greater strength, which either prolongs the time required for its full formation or delays its disruption by the shear forces of sonication.

At 0.8, 0.9 and 1.0 wt% CCNF, thicker physically crosslinked layers form at the onset of sonication due to increased nanofibril density, enhanced hydrogen bonding and secondary interactions. Ultrasonic cavitation mobilizes chains, increasing their specific surface area and enabling rapid network formation. These layers decreased sharply within 30 s, with steeper declines at 0.9 and 1.0 wt% (Fig. 6), as denser networks may form less homogeneous structures that are more susceptible to shear forces from collapsing cavitation bubbles. The quaternary ammonium groups’ permanent positive charge enhances electrostatic and ionic interactions, promoting rapid, thick layer formation, but potentially introduces structural fragility under prolonged sonication.

For the 0.8 wt% solution, two subsequent increases in crosslinked layer height occur at 80 and 280 s, driven by cavitation-induced chain realignment, followed by steady thinning as shear forces dominate. The second increase results in a layer thickness comparable to that of the 0.9 and 1.0 wt% solutions. These higher-concentrated solutions exhibit similar behavior: an initial sharp decrease, a rapid increase at approximately 45 s due to renewed physical crosslinking, and a subsequent steady decline.

The 1.0 wt% solution shows fewer fluctuations, indicating greater network stability due to higher nanofibril density and stronger cationic interactions. With the 0.9 and 1.0 wt% CCNF solutions, significant part of the layer also remains detectable even at the end of sonication. Here the remaining part for the 1.0 wt% sample (7.6% visible height) is approximately twice as thick as that for the 0.9 wt% sample (4.3% visible height). This persistence reflects robust intermolecular interactions that resist complete disruption at higher concentrations. We can conclude that increasing the concentration of CCNF accelerates hydrogel network formation, enhances layer thickness and stability, and delays or prevents complete collapse under sonication. At 1.0 wt%, the CCNF content ensures sufficient interaction strength to maintain partial network integrity despite cavitation-induced shear.

For the 2.0 wt% CCNF sample, a decrease in physically crosslinked layer height was also observed in the first 30 s of sonication as ultrasonic cavitation disrupts intermolecular interactions and fragments nanofibrils, increasing their specific surface area (Fig. 6a). From 30 to 210 s, the layer height fluctuates between 66% and 81% of the visible solution, reflecting a balance between cavitation-induced shear forces breaking weak bonds and chain mobilization promoting new interactions. Between 210 and 400 s, alternating increases and decreases in layer height occur, with a general trend toward an increasing physically crosslinked area, driven by enhanced availability of surface groups that strengthen the network. After 400 s, the layer stabilizes at approximately 90% height, indicating a highly dense network that resists further disruption by cavitation-induced shear. At 2 wt% CCNF, the presence of a developed hydrogel network is evident from the onset of sonication. The persistent high crosslink density at 2.0 wt% underscores the ability of cationic interactions to maintain structural integrity under prolonged sonication.

In contrast, TOCNF solutions at 1.0–1.5 wt% (Fig. 6b) exhibit limited physical crosslinking due to electrostatic repulsion from anionic carboxylate groups, which hinders nanofibril interactions at lower concentrations. No crosslinked layers formed in the 1.0 and 1.2 wt% solutions, as the number of available surface groups is insufficient to overcome repulsion and sustain a stable hydrogel network. At 1.1 wt%, a transient, shallow physically crosslinked layer forms around 30 s, likely a statistical outlier, as cavitation temporarily enables weak hydrogen bonding. Such behavior was consistently observed even after three experimental repetitions, leading us to conclude that it is most likely caused by the dispersion’s physical properties and not experimental error.

The intermittent appearance of a minimal crosslinked layer is most likely due to the fact that the experiment was conducted on a sample containing the minimum concentration of TOCNF required for swelling. Below this threshold, the number of surface substituent groups is insufficient to form enough interactions between polymer chains to sustain a stable hydrogel network. At 1.3, 1.4, and 1.5 wt% (Fig. 6b), physically crosslinked layers form within seconds and persist up to approximately 100 s, driven by increased nanofibril density, reducing repulsion. These layers, however, disintegrate completely due to weaker intermolecular anionic interactions compared to CCNF, with cavitation-induced shear disrupting the less stable networks. The rapid collapse and absence of physical re-crosslinking highlight the limited network stability of TOCNF at these concentrations, contrasting with the persistent layers in CCNF.

Nevertheless, sonication of 1.3, 1.4 and 1.5 wt% TOCNF solutions (Fig. 6b) reveals consistent physical crosslinking behavior. No crosslinking occurs prior to sonication, as repulsion between carboxylate groups prevents spontaneous network formation. Physically crosslinked layers formed within 30 s due to sufficient nanofibril density. However, these layers degraded at a similar rate as they appeared, with the 1.3 and 1.4 wt% samples exhibiting nearly identical thickness profiles. For the 1.3 wt% sample, a shallow secondary crosslinked layer emerged at approximately 70 s, immediately after the initial layer’s collapse.

Similarly, the 1.5 wt% solution exhibited a brief increase in layer thickness at around 60 s, peaking at 70 s before rapidly degrading completely, reflecting a slightly higher nanofibril density enabling short-term network reorganization. These secondary peaks suggest that carboxylate groups facilitate transient re-gelation, but their anionic repulsion limits network stability under prolonged sonication, leading to complete layer disintegration. Minor traces of physical crosslinking detected thereafter are likely artifacts of analysis variability, as persistent networks are not sustained at these concentrations.

Similar to the case of CCNF, increasing the polymer concentration of TOCNF leads to an additional effect during sonication. This can be attributed to the rise in polymer content reaching a threshold where the modified functional groups, specifically carboxyl (COOH) groups, become sufficiently abundant to significantly contribute to physical crosslinking and the formation of the hydrogel network. Increasing TOCNF concentration enhances the contribution of carboxylate groups, reaching a threshold at 1.3–1.5 wt% where hydrogen bonding and ionic interactions support rapid initial gelation. However, unlike CCNF’s robust cationic networks, TOCNF’s weaker interactions result in faster degradation, highlighting the critical role of surface chemistry in hydrogel stability.

For the 2.0 wt% TOCNF sample, the physical crosslinking trend mirrors that of the 2.0 wt% CCNF sample, with a gradual increase in crosslinked area throughout sonication (Fig. 6). Unlike CCNF, which exhibits an initial drop in crosslinked layer height, the TOCNF sample shows no such decrease. In the latter, cavitation-induced chain mobilization immediately enhances hydrogen bonding and ionic interactions without disrupting pre-formed networks. From 30 to 165 s, the physically crosslinked area fluctuates between 60% and 72% of the visible solution, reflecting dynamic competition between cavitation-induced shear forces and network reformation. After 165 s, a steady increase in crosslinked area begins, stabilizing at approximately 85% with minor variations until sonication ends. The absence of an initial drop and the high final crosslinked area highlight the critical role of concentration in enabling TOCNF gelation despite anionic repulsion.

The morphologically divergent growth behaviors of the 2.0 wt% CCNF and TOCNF layers reveal contrasting structural reorganization pathways under identical hydrodynamic fields. The initial reduction in the CCNF crosslinked layer volume indicates a shear-induced compaction mechanism. Because CCNF forms spontaneous attractive physical junctions, the onset of high-intensity ultrasonic shear disrupts peripheral contacts, prompting the internal network to reorient into a tighter, structurally dense configuration. Subsequent layer expansion is driven by ongoing cavitation-induced fibril fragmentation, which exposes hidden quaternary ammonium groups that propagate new crosslinks outward. Conversely, TOCNF exhibits a strictly monotonic increase in layer volume because it lacks a pre-existing attractive framework. The continuous growth of the TOCNF layer represents a pure steady-state accumulation process, where continuous acoustic streaming repeatedly forces mutually repulsive anionic fibrils to overcome their electrostatic double-layer barriers, trapping them step-by-step into a progressive hydrogen-bonded network.

We can conclude that both modified forms, CCNF and TOCNF, show similar trends with increasing sonication time, however, the underlying polymer chain dynamics differ. At equal concentrations and without a crosslinking agent, their ability to form hydrogels diverges, primarily due to contrasting surface chemistry. CCNF carry positive charges, which promote attractive interactions between fibrils, leading to faster network formation and hydrogel development even at lower concentrations, with a minimum threshold of approximately 0.5 wt% for swellable networks. This leads to denser, more heterogeneous hydrogels that form early and resist complete disruption under cavitation-induced shear. In contrast, TOCNF’s anionic carboxylate groups, introduced via TEMPO oxidation, induce strong electrostatic repulsion between fibrils, thereby stabilizing the dispersion and hindering gelation, thus requiring higher concentrations (≥1.3 wt%) to achieve sufficient fibrillar crowding for network formation. Consequently, CCNF hydrogels tend to be denser and more heterogeneous, while TOCNF hydrogels are more open and uniform but less stable, with weaker networks that collapse rapidly under prolonged sonication. At 2.0 wt%, both systems achieve comparable physically crosslinked layer thicknesses, as high nanofibril density mitigates the influence of surface charge differences, enabling robust gelation. These findings underscore that CCNF’s cationic interactions facilitate earlier and stronger crosslinking, while TOCNF’s anionic repulsion necessitates higher concentrations for equivalent network stability.

## 4 Conclusions

This study provides the first direct, real-time visualization of hydrogel network formation in nanocellulose dispersions during sonication, highlighting the critical influence of both concentration and nanofibril surface chemistry on dynamic gelation behavior. Through imaging, statistical evaluation and particle image velocimetry (PIV), we systematically investigated the sonication response of cationic (CCNF) and TEMPO-oxidized (TOCNF) cellulose nanofibrils across a broad concentration range.

The results reveal that CCNF dispersions undergo rapid physical crosslinking even at low concentrations (≥0.5 wt%), forming persistent hydrogel networks due to strong attractive ionic interactions between quaternary ammonium groups. These networks exhibit high resistance to ultrasonic disruption and remained stable throughout sonication, particularly at higher concentrations (≥0.9 wt%). In contrast, TOCNF dispersions require higher concentrations (≥1.3 wt%) to overcome electrostatic repulsion between carboxylate groups and form hydrogel networks. However, these structures are transient and degrade more rapidly under acoustic shear.

Increasing nanofibril concentrations enhanced gelation kinetics, increased crosslinked layer thickness and improved structural stability in both CCNF and TOCNF systems. At 2.0 wt%, both nanocellulose types formed extensive hydrogel networks. However, CCNF consistently achieved greater physical crosslink density, thicker and more stable networks and stronger resistance to cavitation-induced degradation compared to TOCNF.

By linking nanofibril surface functionality with real-time network dynamics under sonication, this work offers mechanistic insight into the formation and stability of nanocellulose-based hydrogels. With this it demonstrates that surface chemistry and concentration are the dominant parameters governing gelation kinetics, hydrogel network stability and resistance to ultrasonic disruption in nanocellulose dispersions. This work provides important mechanistic insight into ultrasound-induced gelation and establishes a methodological framework for the development of nanocellulose-based hydrogels for advanced applications, including biomedical scaffolds, 3D printing inks, and smart rheological modifiers.

Due to the novelty of the high-speed characterization approach, the study focused primarily on the influence of selected ultrasonic parameters, neglecting for example the influence of pH, temperature and ultrasonic power. Future studies should therefore examine the combined influence of processing and environmental parameters under application relevant conditions.

## CRediT authorship contribution statement

**Žan Boček:** Conceptualization, Data curation, Investigation, Methodology, Visualization, Writing – original draft, Writing – review & editing. **Tilen Kopač:** Conceptualization, Investigation, Methodology, Writing – original draft, Writing – review & editing. **Aleš Ručigaj:** Conceptualization, Investigation, Methodology, Writing – original draft, Writing – review & editing, Data curation, Supervision. **Matevž Dular:** Conceptualization, Funding acquisition, Investigation, Methodology, Resources, Writing – original draft, Writing – review & editing.

## Declaration of competing interest

The authors declare that they have no known competing financial interests or personal relationships that could have appeared to influence the work reported in this paper.

## Data Availability

Data will be made available on request.

## Appendix A Supplementary data

Supplementary video data of nanofibril solution sonication. The following are the Supplementary data to this article:
